# Carney Complex During Six-Year Follow-Up and Its Association With Attention-Deficit Hyperactivity Disorder: A Case Report

**DOI:** 10.7759/cureus.94953

**Published:** 2025-10-19

**Authors:** Qian-Hui Tai, Chun-Yang Li, Qian-Long Liu, Zhe Yang, Wei Cui

**Affiliations:** 1 Department of Endocrinology, The First Affiliated Hospital of Xi'an Jiaotong University, Xi'an, CHN; 2 Department of Child Health Care, Xi’an Children’s Hospital, Xi'an, CHN; 3 Department of Pediatric Surgery, The Second Affiliated Hospital of Xi'an Jiaotong University, Xi'an, CHN; 4 Department of Pathology, The First Affiliated Hospital of Xi'an Jiaotong University, Xi'an, CHN

**Keywords:** adrenal gland, attention-deficit hyperactivity disorder, carney complex, cushing’s syndrome, precocious pubert

## Abstract

Carney complex is a rare autosomal dominant genetic syndrome that may involve multiple endocrine glands. As the disease progresses, different symptoms gradually emerge. Follow-up is crucial for early diagnosis and treatment of each neoplasm. Case report: A 3.8-year-old Han Chinese male patient presented with acne for one year. He was diagnosed with Carney complex for Cushing's syndrome caused by an adrenal adenoma, precocious puberty mainly caused by a large cell calcifying Sertoli cell tumour of the testis. Left adrenalectomy and a treatment for precocious puberty, including aromatase inhibitor therapy, were prescribed. The patient experienced complete relief from Cushing's syndrome and showed an improvement in predicted adult height. Attention-deficit hyperactivity disorder impaired the patient's attention. Both the patient and his parents were receiving behavior management training. Carney complex patients need close follow-up. Adrenal adenoma is also a possible pathological type of adrenal lesion in this complex. Multidrug combination therapy could have a good effect on precocious puberty. Attention-deficit hyperactivity disorder was present as a comorbid condition, the underlying mechanism of which in patients with Carney complex requires further investigation. Collaboration among multidisciplinary teams is crucial for prognosis.

## Introduction

Carney complex (CNC) is a rare autosomal dominant genetic syndrome characterized by skin and mucosal pigmentation, cardiac myxomas, and multiple endocrine tumors [1]. Its prevalence remains unknown. In the largest genotyped patient series reported, 63% were female and 37% were male [2]. The disease is primarily caused by inactivating mutations or large deletions in the PRKAR1A gene, which encodes the regulatory subunit (RⅠα) of protein kinase A (PKA) located at 17q22-24. These genetic alterations lead to unregulated catalytic subunit activity, increased cell proliferation in cAMP-responsive tissues, and subsequent tumor formation. A minority of cases are associated with other genetic abnormalities, including copy number gains in PRKACA or PRKACB, or mutations in the 2p16 region [2,3].

In CNC, each specific complication should be addressed separately. Due to the rarity of the disease, understanding of its full clinical spectrum continues to evolve through ongoing case reports. Current management remains largely recommendation-based owing to limited evidence. For instance, non-surgical approaches are preferred for testicular tumors, with aromatase inhibitors reserved for use when necessary [4]. However, specific protocols regarding treatment duration and response evaluation are lacking, which poses considerable challenges in clinical decision-making.

In this report, we present a six-year follow-up record of a CNC patient admitted to our center. Notably, we describe two uncommon manifestations: Cushing's syndrome caused by an adrenal adenoma and comorbid attention-deficit hyperactivity disorder (ADHD), both rarely documented in CNC. Additionally, we provide a detailed account of the integrated management and therapeutic response of testicular tumors in this patient, which may serve as a valuable reference for clinicians managing similar cases.

## Case presentation

**Figure 1:**
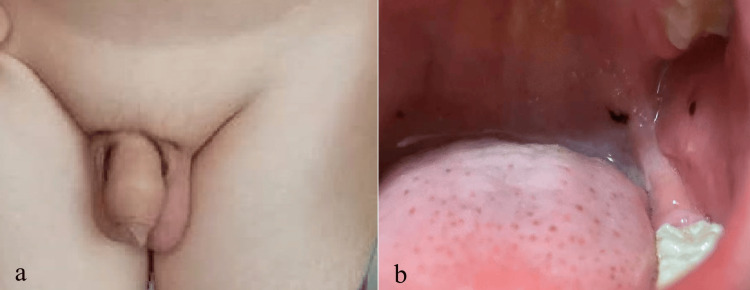
The patient's physical characteristics (a) External genital development of the patient at his first admission. (b) The patient had a blue nevus on the oral mucosa at the age of six years and four months.

A three-year-and-10-month-old Han Chinese boy was admitted to our hospital in July 2019 for the first time, and complained of having acne for one year. Physical examination revealed that the boy was 109 cm tall and weighed 20 kg (Table 1). He has acne on the face, neck, and chest, and increased vellus hair on the face, back, limbs, and genital area. His external genital development reached Tanner genital stage II (G2) (Figure 1a). His mother denied the application history of glucocorticoid medicines and any other long-term medication history of the boy. At that initial examination, no lesions were observed on the oral mucosa. During regular follow-up over the subsequent year (at age six years and four months), blue nevus emerged on his buccal mucosa, and these new lesions progressively increased in size and number (Figure 1b).

**Table 1:** Laboratory examinations and vitals at first admission ACTH, Adrenocorticotropic hormone. "NA" indicates parameters with no established reference ranges. A hyphen ("-") denotes data not measured or inherently empty fields in the format. *Reference ranges were based on the growth standard for children under seven years in China [5].

Parameter	Result	Unit	Reference range
Height	109	cm	Three years nine month boys (P10: 98.3cm, P90: 108.0cm)*
Weight	20	Kg	Three years nine month boys (P10: 14.1kg, P90: 18.7kg)*
Cortisol levels	-	-	-
At 8:00	28.7	μg/dL	5.0-28.0
At 16:00	25.1	μg/dL	NA
At 8:00	26.7	μg/dL	NA
ACTH at 8:00	2.2	Pg/mL	7.2-63.3
A high-dose 2-day dexamethasone test	-	-	-
Cortisol level at 8:00 am on the first day	34.6	μg/dL	5.0-28.0
ACTH level at 8:00 am on the first day	2.9	pg/mL	7.2-63.3
Cortisol level at 8:00 am on the third day	47.0	μg/dL	NA
ACTH level at 8:00 am on the third day	2.1	pg/mL	NA

**Figure 2:**
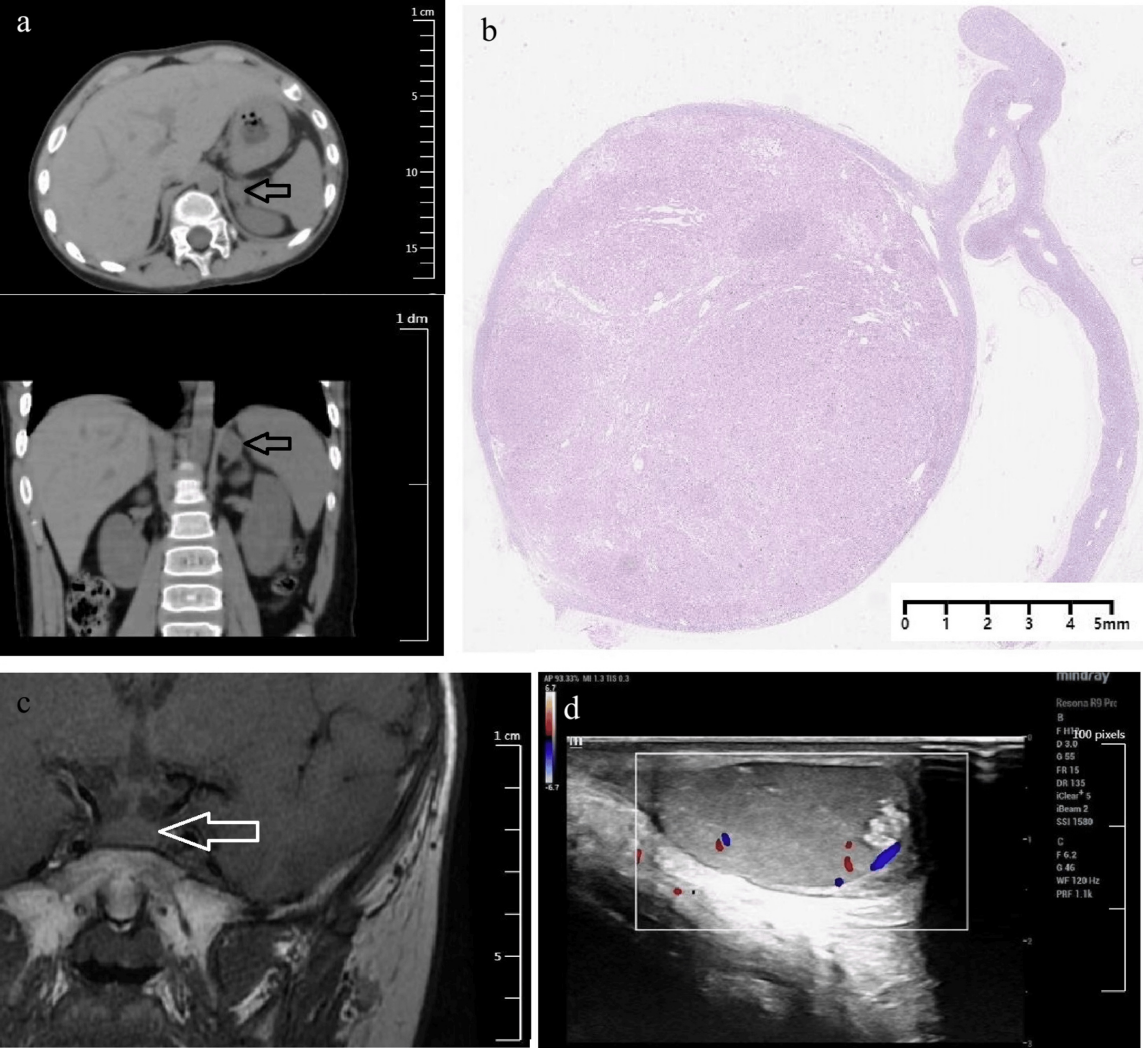
Representative imaging and pathologic findings of multifocal lesions in the adrenal gland, pituitary, and testis (a) Adrenal CT scan images. (b) Pathology of the left adrenal. Immunological parameters: CK (-), EMA (-), Vim (-), CgA (-), SyN (+), CD56 (-), S-100 (-), Ki67 (+) 3%. (c) Pituitary MR image. (d) Testis ultrasound image.

The patient's father was diagnosed with cerebral infarction, left atrial myxoma and a bicuspid aortic valve. The boy's grandfather underwent myxoma resection surgery twice, at the ages of 53 and 55. The patient's uncle and cousins did not have related symptoms. The patient had a high level of serum cortisol and lost the normal circadian rhythm of cortisol secretion. At 8:00, 16:00, and 24:00, his cortisol levels were 28.7μg/dl, 25.1μg/dl and 26.7μg/dl, respectively. In a high-dose two-day dexamethasone test, the serum cortisol level was 34.6μg/dl at 8:00 am on the first day, while the serum ACTH level was 2.9pg/ml. On the third day at 8:00 am, the serum cortisol level was 47.0μg/dl, while the serum ACTH level was 2.1pg/ml. ACTH was consistently subnormal, whereas the 8:00 am cortisol level on the third day failed to suppress. The results presented in Table 1 indicated ACTH-independent Cushing's syndrome in the patient. Enhanced CT revealed a 13.7mm×20.1mm×19.7mm nodule in the medial branch of the left adrenal gland with a clear border (Figure 2a), and the right adrenal gland was normal in shape. In fact, pathological examination following the subsequent left adrenalectomy (performed at the age of three years and nine months) revealed an adrenal adenoma, as demonstrated in Figure 2b. Pituitary MRI showed a slightly enlarged pituitary gland, especially at the upper edge, with a height of 5.6mm, and there were no obvious abnormal signals inside (Figure 2c). Bilateral testicular MRI signals are uneven, with slightly low signal shadows visible in T2. Ultrasound showed hypoechoic nodules in both testicles, with scattered strong echoic spots in the left testicle (Figure 2d). Ultrasound of the thyroid and heart was normal. The patient's genetic testing revealed a suspected heterozygous deletion mutation in the chr17:66511508-66551912 region. On the basis of the clinical manifestations, family history of cardiac myxoma, laboratory tests, imaging examinations, and genetic testing, the boy was diagnosed with Carney complex according to the diagnostic criteria [2]. One and a half months after the left adrenalectomy, the patient's body weight decreased by 1.2kg. His body shape and appearance returned to normal. Serum cortisol and ACTH returned to normal levels. The patient's mother was satisfied with the treatment effect.

**Figure 3:**
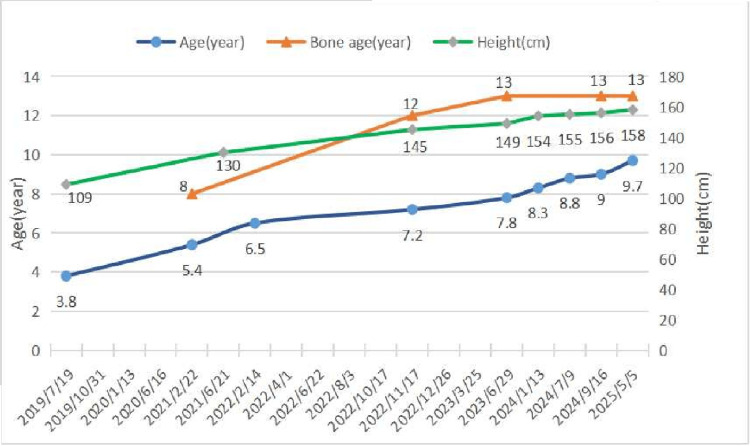
Line chart of the patient's age, bone age, and height

When he was five years and eight months old, he had nodular tissue on his neck and underwent surgical resection. The pathological diagnosis was "chronic inflammation with fibromatous hyperplasia". When the boy was six years and five months old, a left testis biopsy was performed. The pathological diagnosis was consistent with large cell calcifying Sertoli cell tumours of the testis (LCCSCTs). The Immunohistochemical results were as follows: Inhibin-a (+), Ki-67 (5%+), Calletinin (+), Melan-A (+), CK (AE1/AE3) (occasional+), CD99 (partial+), PLAP (-), Masson (-). He had a bone age advanced by more than two years, with developing external genitalia and significantly elevated testosterone levels. Then he received a treatment for precocious puberty. Table 2 and Figure 3 show the patient's sex hormones, growth hormones, height, bone age, and medication treatment during the follow-up period. The data indicate suppression of Luteinizing Hormone (LH), Follicle-Stimulating Hormone (FSH), and testosterone to relatively low levels and a decelerated rate of bone age advancement.

**Table 2:** Changes in age, height, bone age, sex hormones, and growth hormone levels of the patient during the follow-up IGFBP-3, Insulin-like growth factor binding protein-3; GH, growth hormone; DHEAS, Dehydroepiandrosterone sulfat; E2, Estradiol; P, Progesterone; PRL, Prolactin; TT, total Testosterone; IGF-1, Insulin-like growth factor-1. *The patient started receiving treatment for precocious puberty in March 2022. A hyphen ("-") denotes data not measured or inherently empty fields in the format. Reference range^a^ is for males ≤ 7.1 years old. However, the ranges for growth hormone (GH), Insulin-like growth factor 1 (IGF-1), and Insulin-like growth factor binding protein (IGFBP-3) within this group are specifically for males between four and six years old. Reference range^b^ is for males between 7.1 and 12.1 years old. However, the ranges for GH, IGF-1, and IGFBP-3 within this group are specifically for males between seven and 10 years old. Males under 7.1 years are usually in Tanner Stage I. Males between 7.1 and 12.1 years are usually in Tanner Stage II. Reference ranges were based on the standards from our hospital laboratory and published multicenter data for Chinese children [6].

Age (year)	Bone age (year)	Height (cm)	E2 (pmol/l)	P (nmol/l)	PRL (ng/ml)	LH (mIU/ml)	FSH (mIU/ml)	TT (nmol/l)	DHEAS (umol/l)	GH (ng/ml）	IGFBP-3 (ug/ml)	IGF-1 (ug/ml)	Time
3.8	-	109	＜18.4	1.85	25.74	＜0.1	0.311	9.5	1.54	1.93	-	-	2019/7/19
4.1	-	-	＜18.4	0.31	18.33	0.367	0.418	5	0.077	-	-	-	2019/10/31
4.3	-	-	＜18.4	0.37	8.11	0.31	0.671	4.76	0.302	-	-	-	2020/1/13
4.7	-	-	34.2	0.18	8.18	0.199	0.711	5.62	0.269	-	-	-	2020/6/16
5.4	8	-	24	＜0.159	9.78	0.234	0.425	8.6	0.262	-	-	-	2021/2/22
5.8	-	-	-	-	-	-	-	7.82	-	-	-	-	2021/6/21
6.4	-	-	＜18.4	＜0.159	10.98	＜0.1	0.179	10.32	0.294	-	-	-	2022/2/14*
6.8	-	-	＜18.4	0.627	10.34	0.368	0.156	4.16	0.227	-	-	-	2022/6/22
6.9	-	-	66.5	0.827	6.26	0.733	1.61	4.06		-	6.68	292	2022/8/3
7.1	-	-	-	-	-	-	-	4.18	-	-	-	-	2022/10/17
Reference range^a^	-	-	≤47.7	≤1.59	4.5 - 18	0.09 - 0.43	0.06 - 3.27	0.1 - 1.12	0.01 - 0.53	0.09 - 2.5	1.3 - 5.6	22 - 208	-
7.2	12	145	70.9	0.598	7.58	0.274	＜0.1	3.59	0.54	-	-	-	2022/11/17
7.3	-	-	58.6	0.655	8.41	＜0.1	0.179	4.29	0.311	-	-	-	2022/12/26
7.5	-	-	55.5	0.337	8.76	＜0.1	0.172	3.81	0.242	-	-	-	2023/3/25
7.8	13	149	＜18.4	＜0.159	7.88	＜0.1	0.233	4.97	0.396	10.6	6.48	382	2023/6/29
8.3	-	154	＜18.4	0.393	10.53	0.136	＜0.1	5.22	0.3	-	-	-	2024/1/13
9.6	13	159	＜18.4	0.458	8.08	0.239	＜0.3	10.35	0.869	6.93	8.56	421	2025/5/5
Reference range^b^	-	-	≤58.7	≤1.59	2.5 - 24.8	0.09 - 4.24	0.12- 3.89	0.1 - 2.37	0.08 - 2.31	0.09 - 3.2	1.8 - 7.1	40 - 255	-

**Figure 4:**
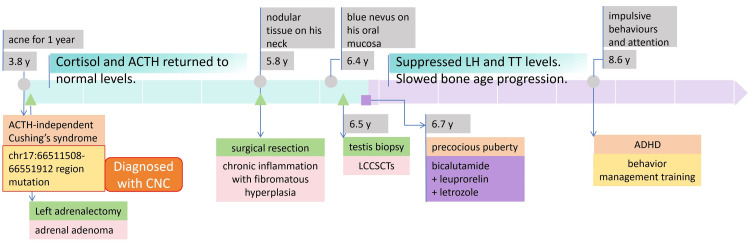
Diagnostic and therapeutic timeline This timeline illustrates the chronological sequence of key clinical events, diagnostic findings, therapeutic interventions and critical laboratory parameters from initial presentation to the last follow-up. Grey circles indicate major clinical events. Green triangles represent surgical procedures or pathological biopsies. The purple rectangle denotes pharmacologic therapies. ACTH, Adrenocorticotropic Hormone; ADHD, attention-deficit hyperactivity disorder; CNC, Carney complex; LCCSCTs, large cell calcifying Sertoli cell tumors; LH, Luteinizing Hormone; TT, total Testosterone.

In primary school, the boy struggled to follow class discipline due to his impulsive behavior and attention deficit. In the clinic, he exhibited hyperactivity, demonstrated by an inability to remain seated, and displayed signs of inattention, including not responding to direct questions. At the age of eight years and six months, the boy underwent a careful consultation and a structured neurobehavioral evaluation comprising a series of tests. The Conners' Parent Rating Scale [7] was administered to screen for attention-deficit hyperactivity disorder (ADHD). As detailed in Table 3, the results revealed clinically significant scores in learning problems (2.8) and impulsive-hyperactive (2.5) domains. According to the clinical data and assisted examination, the patient fulfilled the DSM-5 diagnostic criteria for ADHD [8], combined presentation, with 9/9 inattentive and 8/9 hyperactive-impulsive symptoms. The Wechsler Intelligence Scale for Children (WISC) [9] yielded a full-scale IQ of 86. This comprehensive assessment confirmed the diagnosis of ADHD, leading to the initiation of behavior management training for the family. Figure 4 presents the timeline of the patient's key clinical symptoms, diagnoses, therapeutic interventions, and critical laboratory parameters from initial presentation to the last follow-up.

**Table 3:** Questionnaires used for the evaluation of the case DSM-V, Diagnostic and Statistical Manual of Mental Disorders Version V; WISC, Wechsler Intelligence Scale for Children (4th ed.); ADHD, Attention-Deficit Hyperactivity Disorder. The table combines information from three sources [7-9], each corresponding to a different section of the table.

Test	Results
Conners' Parent Rating Scale	Conduct 1.6
	Learning 2.8
	Psychosomatic 0
	Impulsive-hyperactive 2.5
	Anxiety problems 0.3
	Hyperactivity index 2.2
DSM-V of ADHD	Attention deficit 9/9
	impulsive-hyperactive 8/9
WISC	86

## Discussion

CNC is a rare autosomal dominant genetic syndrome that may involve multiple endocrine glands. Primary pigmented micronodular adrenocortical disease (PPNAD) is a characteristic of CNC. However, recent genomic studies reported that PKA pathway mutations, such as PRKACA, PRKACB, PRKAR1A, and PRKAR1B mutations, are correlated with cortisol-producing adenoma in addition to PPNADs [10]. Our case supports this finding. We showed an adrenal adenoma causing Cushing's syndrome, which was resolved after unilateral adrenalectomy. Collectively, these findings suggest that CNC may encompass a broader spectrum of adrenal diseases. During follow-up, contralateral adrenal gland CT could be performed to monitor for potential hyperplasia or newly developed nodules.

Large cell calcifying Sertoli cell tumours of the testis (LCCSCTs) are estimated to occur in about 50-75% of men with CNC, and are thought to primarily exhibit excessive aromatase activity, leading to the over-conversion of androgens into estrogens, which cause advanced bone age, gynecomastia, peripheral precocious puberty, and, even over time, may induce central precocious puberty. The use of aromatase inhibitors benefits the patients with LCCSCT [11-13]. Nevertheless, the excessive testosterone associated with LCCSCTs has not been explicitly mentioned in previous reports. In our case, after unilateral adrenalectomy, the patient's testosterone level decreased, but was still higher than that of boys before puberty [14], despite normal cortisol, ACTH, and DHEA-S levels, suggesting testicular origin. Pathologically confirmed Leydig cell hyperplasia further supported excess testosterone secreted from damaged testicular tissue, complicating puberty suppression. The patient thus initiated combined therapy (aromatase inhibitor, GnRHa, and antiandrogen) at age 6.5 years to mitigate precocious puberty progression and optimize adult height.

LCCSCTs are mostly benign in children, and only malignant tumors require surgical treatment [12]. The behaviour of LCCSCTs is borderline in cases that feature malignancy, including a maximum diameter of the tumor >4 cm, tumor necrosis, lymphovascular invasion, extratesticular extension, and an elevated mitotic rate [15]. In our case, based on pathological and imaging examinations in follow-up, the patient's testicular tumor does not exhibit malignant features. Medical therapy remains the preferred approach over orchiectomy. However, regular testicular ultrasound surveillance is recommended for LCCSCTs to monitor disease progression.

The patient has received bicalutamide and leuprorelin for 37 months and letrozole for 20 months. His predicted adult height (PAH) increased from 174.3 cm to 180.4 cm (corrected midparental height: 176.5 cm), with slowed bone age progression (Figure 3), confirming treatment efficacy. However, due to his family's financial constraints, therapy was nearly discontinued despite no signs of central precocious puberty. Currently 158 cm tall with a bone age of 13 years (four years advanced), early termination of treatment risks rapid skeletal maturation and epiphyseal closure. Could short-term adjunctive recombinant human growth hormone (rhGH) help achieve an ideal adult height? Indeed, approximately 10% of CNC patients develop acromegaly [2], while this patient exhibited mildly elevated growth hormone levels. The observed growth hormone elevation does not appear to confer additional risk of tumor formation in this clinical context. Importantly, while rhGH therapy shows no proven neoplastic risk in general populations, its safety in genetic predisposition syndromes like CNC remains completely unestablished [16]. Given the lack of data in high-risk cohorts, this approach should be considered strictly experimental, requiring rigorous risk-benefit evaluation and long-term monitoring. 

ADHD significantly impairs the patient's daily life. While ADHD involves genetic and epigenetic dysregulation in neurotransmitter and neurodevelopmental pathways, no association has been established between ADHD and either PRKAR1A or the patient's additional variants of uncertain significance (VUS). Large-scale prospective genomic studies are needed for clearer insights [17].

The role of androgens and aromatase inhibitors in ADHD remains debated. An increased level of prenatal testosterone increases the risk of developing ADHD-like symptoms in offspring [18]. A study found that increased medial temporal lobe and striatal gray matter volumes were associated with abnormal attentional or memory processing in patients who had excess androgen at a young age [19]. ADHD patients had lower dehydroepiandrosterone sulfate (DHEAS) levels [20]. Nevertheless, causal evidence is lacking. Aromatase inhibitors may impair cognition and increase risk-taking behavior, possibly mediated by letrozole's blood-brain barrier permeability and its interaction with central estrogen receptors [21,22]. 

Notably, the core molecular defect in CNC-overactivation of the PKA/cAMP signaling pathway due to PRKAR1A mutation offers a plausible biological link. The co-occurrence of ADHD in this CNC patient may not be coincidental. Precise cAMP/PKA signaling in the prefrontal cortex and striatum is crucial for attention and behavioral control. The ADHD medication guanfacine acts by suppressing cAMP production, while dysregulation of the dopamine/cAMP/PKA pathway in the striatum directly leads to hyperactivity and impulsivity in animal models [23,24]. Thus, we hypothesize that the patient's PRKAR1A mutation may contribute to his ADHD phenotype by disrupting cAMP/PKA homeostasis in the central nervous system. However, further studies are required to determine whether ADHD is a potential neurological manifestation of the CNC spectrum, a therapy side effect, or an independent comorbidity.

## Conclusions

This case details a six-year follow-up of a pediatric patient with Carney complex (CNC), highlighting three key findings that expand the current understanding of the disease spectrum. First, while primary pigmented nodular adrenocortical disease (PPNAD) has been regarded as the characteristic adrenal manifestation of CNC, our case demonstrates that an adrenal adenoma can also cause Cushing's syndrome in this context. Second, for precocious puberty associated with large cell calcifying Sertoli cell tumors (LCCSCTs), the multidrug combination therapy demonstrated a favorable outcome, as evidenced by the notable improvement in the patient's predicted adult height and the effective control of bone age progression. Third, clinicians should be aware of a potential association with ADHD in CNC patients, a link that remains uncharacterized but clinically important. These unique features underscore the importance of individualized multidisciplinary management and further mechanistic research into the broad phenotypic variability of CNC.

## References

[1] Stratakis CA (2003). Carney Complex..

[2] Correa R, Salpea P, Stratakis CA (2015). Carney complex: an update. Eur J Endocrinol.

[3] Wilkes D, McDermott DA, Basson CT (2005). Clinical phenotypes and molecular genetic mechanisms of Carney complex. Lancet Oncol.

[4] Wasserman JD, Schneider KW, Achatz MI (2025). Updated recommendations for pediatric surveillance in hereditary endocrine neoplasia syndromes: multiple endocrine neoplasias, hyperparathyroidism-jaw tumor syndrome, and carney complex. Clin Cancer Res.

[5] (2025). National Health Commission of the People's Republic of China. Growth Standard for Children Under 7 Years: WS/T 423—2022 [S/OL]. https://www.nhc.gov.cn/fzs/c100048/202211/5001d7cf57774770a1d49c1df46a291f.shtml.

[6] Zhong X, Ding J, Zhou J (2018). A multicenter study of reference intervals for 15 laboratory parameters in Chinese children (Article in Chinese). Zhonghua Er Ke Za Zhi.

[7] Conners CK, Sitarenios G, Parker JD, Epstein JN (1998). The revised Conners' Parent Rating Scale (CPRS-R): factor structure, reliability, and criterion validity. J Abnorm Child Psychol.

[8] American Psychiatric Association (2013). Diagnostic and Statistical Manual of Mental Disorders, Fifth Edition. https://psychiatryonline.org/doi/book/10.1176/appi.books.9780890425596.

[9] Wechsler D (2003). Wechsler Intelligence Scale for Children (4th ed.).

[10] Faillot S, Foulonneau T, Néou M (2021). Genomic classification of benign adrenocortical lesions. Endocr Relat Cancer.

[11] Dağdeviren Çakır A, Turan H, Celkan T, Çomunoğlu N, Ercan O, Evliyaoğlu O (2020). An unusual presentation of Carney complex. J Clin Res Pediatr Endocrinol.

[12] Gourgari E, Saloustros E, Stratakis CA (2012). Large-cell calcifying Sertoli cell tumors of the testes in pediatrics. Curr Opin Pediatr.

[13] Crocker MK, Gourgari E, Lodish M, Stratakis CA (2014). Use of aromatase inhibitors in large cell calcifying sertoli cell tumors: effects on gynecomastia, growth velocity, and bone age. J Clin Endocrinol Metab.

[14] Wei C, Davis N, Honour J, Crowne E (2017). The investigation of children and adolescents with abnormalities of pubertal timing. Ann Clin Biochem.

[15] Al-Obaidy KI, Idrees MT, Abdulfatah E, Kunju LP, Wu A, Ulbright TM (2022). Large cell calcifying Sertoli cell tumor: a clinicopathologic study of 18 cases with comprehensive review of the literature and reappraisal of prognostic features. Am J Surg Pathol.

[16] Bamba V, Kanakatti Shankar R (2022). Approach to the Patient: Safety of Growth Hormone Replacement in Children and Adolescents. J Clin Endocrinol Metab.

[17] Thapar A, Cooper M (2016). Attention deficit hyperactivity disorder. Lancet.

[18] Dinkelbach L, Peters T, Grasemann C, Hebebrand J, Hinney A, Hirtz R (2024). No evidence for a causal contribution of bioavailable testosterone to ADHD in sex-combined and sex-specific two-sample Mendelian randomization studies. Eur Child Adolesc Psychiatry.

[19] Mueller SC, Merke DP, Leschek EW, Fromm S, VanRyzin C, Ernst M (2011). Increased medial temporal lobe and striatal grey-matter volume in a rare disorder of androgen excess: a voxel-based morphometry (VBM) study. Int J Neuropsychopharmacol.

[20] Wang LJ, Lee SY, Chou MC, Lee MJ, Chou WJ (2019). Dehydroepiandrosterone sulfate, free testosterone, and sex hormone-binding globulin on susceptibility to attention-deficit/hyperactivity disorder. Psychoneuroendocrinology.

[21] Goudriaan AE, Lapauw B, Ruige J, Feyen E, Kaufman JM, Brand M, Vingerhoets G (2010). The influence of high-normal testosterone levels on risk-taking in healthy males in a 1-week letrozole administration study. Psychoneuroendocrinology.

[22] Kil KE, Biegon A, Ding YS (2009). Synthesis and PET studies of [(11)C-cyano]letrozole (Femara), an aromatase inhibitor drug. Nucl Med Biol.

[23] Alamo C, López-Muñoz F, Sánchez-García J (2016). Mechanism of action of guanfacine: a postsynaptic differential approach to the treatment of attention deficit hyperactivity disorder (ADHD). Actas Esp Psiquiatr.

[24] Napolitano F, Bonito-Oliva A, Federici M (2010). Role of aberrant striatal dopamine D1 receptor/cAMP/protein kinase A/DARPP32 signaling in the paradoxical calming effect of amphetamine. J Neurosci.

